# Long-term (30 days) toxicity of NiO nanoparticles for adult zebrafish *Danio rerio*

**DOI:** 10.2478/intox-2014-0004

**Published:** 2014-07-16

**Authors:** Jevgenij A. Kovrižnych, Ružena Sotníková, Dagmar Zeljenková, Eva Rollerová, Elena Szabová

**Affiliations:** 1 Slovak Medical University in Bratislava, Limbová 12, 833 03, Bratislava, Slovakia; 2 Institute of Experimental Pharmacology and Toxicology, Slovak Academy of Sciences, Dúbravská 9, 841 04 Bratislava, Slovakia

**Keywords:** zebrafish, toxicity, nickel oxide, nanoparticles

## Abstract

Nickel oxide in the form of nanoparticles (NiO NPs) is extensively used in different industrial branches. In a test on adult zebrafish, the acute toxicity of NiO NPs was shown to be low, however longlasting contact with this compound can lead to its accumulation in the tissues and to increased toxicity. In this work we determined the 30-day toxicity of NiO NPs using a static test for zebrafish *Danio rerio*. We found the 30-day LC_50_ value to be 45.0 mg/L, LC_100_ (minimum concentration causing 100% mortality) was 100.0 mg/L, and LC_0_ (maximum concentration causing no mortality) was 6.25 mg/L for adult individuals of zebrafish. Considering a broad use of Ni in the industry, NiO NPs chronic toxicity may have a negative impact on the population of aquatic organisms and on food web dynamics in aquatic systems.

## Introduction

Nickel oxide (NiO) is one of the important industrial materials used in electronic and ceramic engineering. Nanosized nickel oxide possesses many unique properties compared to its bulk counterpart and is extensively used as catalyzer, battery electrodes, electrochromic films, sensor magnetic materials, printing inks, diesel-fuel additive, *etc.* (Salimi *et al.*, 2007; Rao and Sunandana, 2008; Schrand *et al.*, 2010).

In spite of a broad use of NiO NPs in industry and mainly in diesel fuel, little is known about the potential behavior and ecotoxicity of nanoparticles to aquatic organisms, including fish.

Kovrižnych *et al.* (2013) showed low acute toxicity of NiO nanoparticles for adult individuals of zebrafish (48-h LC_50_=760 mg/L and 96-h LC_50_=420 mg/L) and also low acute toxicity for eggs of zebrafish (96-h LC_50_=1 300 mg/L). NiO NPs did not induce malformations of fish body, yet in the concentrations of 800 and 1 600 mg/L a mild extension of the volume and stretching of the yolk pouch was observed. After 96 hours, 50% of the embryos were born in the control conditions, 20% in 100 mg/L NiO NPs, and 10% in 200 mg/L NiO NPs. However, the nickel oxide nanoparticles induced delayed hatching and in concentrations of 400 and 800 mg/L no embryo was born up to 8 days from the beginning of incubation.

Lin *et al.* (2011) and Lin *et al.* (2013) showed that 4 metal oxide nanoparticles (CuO, ZnO, Cr_2_O_3_, and NiO) could interfere with zebrafish embryo hatching by a chelator-sensitive mechanism that involves ligation of critical histidines in the zebrafish hatching enzyme 1 (ZHE1) center by shed metal ions. A recombinant ZHE1 enzymatic assay was established to demonstrate that the dialysates from the same materials responsible for hatching interference also inhibited ZHE1 activity in a dose-dependent fashion. While neither the embryos nor larvae demonstrated morphological abnormalities, high content fluorescence-based imaging demonstrated that CuO, ZnO, and NiO could induce increased expression of the heat shock protein 70.

By considering other organisms, Gong *et al.* (2011) showed that the NiO NPs had severe impact on algae, with 72-h EC_50_ values of 32.28 mg NiO/L. Under the stress of NiO NPs, Chlorella vulgaris cells showed plasmolysis, cytomembrane breakage and thylakoid disorder.

Hanawa *et al.* (1992) examined the cytotoxic properties of a broad spectrum of metal oxide particles (500–3 000 nm) with respect to fibroblasts (cells were incubated in the presence of particles for 24 hours). The fine NiO NPs did not exert cytotoxic effect. However, Horie *et al.* (2009) found ultrafine NiO to be more cytotoxic than fine NiO *in vitro*. Also Wu (2012) reported that NiO NPs belong to the moderate cytotoxicity category.

Soluble NiCl_2_ and NiO nanoparticles were equally toxic to H460 human lung epithelial cells and primary human bronchial epithelial cells (Pietruska *et al.*, 2011). In rodent inhalation studies, NiO particles (2.2–2.5 µm) induced lung tumors (Dunnick *et al.*, 1995). Inhalation of nickel compounds is an occupational hazard associated with development of human lung, nasal, and paranasal sinus cancers (Straif *et al.*, 2009).

In the majority of studies on NiO NPs toxicity, the authors performed tests with short-term effect of NiO NPs on different organisms and their cells (from several hours to four days). However, only few works (Dunnick *et al.*, 1995; Ogami *et al.*, 2009) described the effect of NiO NPs in chronic toxicity tests on rats. At the same time no work exists which would study the longlasting effect of NiO NPs on fish. Based on the study of Han *et al.* (2012), who used *Gracilaria lemaneiformis* as the model organism, we suggest that NiO NPs can accumulate in fish body. Therefore the aim of the work was to determine chronic (30 days) toxicity of NiO NPs on the adult fish *Danio rerio*.

## Methods

The stock dispersion of the required amount of nickel (II) oxide nanoparticles (<50 nm, 99.8% trace metals basis) from Sigma-Aldrich Chemie GmbH (Germany) was prepared in 100 ml tap water using an ultrasound homogenizer Sonopuls HD 2070. Final concentrations were prepared by dilution of the stock dispersion with tap water. The time of preparation of the stock dispersion NiO NPs was 20 minutes. Animal experiments were performed in the laboratory fulfilling the criteria of Good Laboratory Practice. They were conducted in accordance with the EEC Directive of 1986; 86/609/EEC and approved by the State Veterinary and Food Administration of the Slovak Republic. Tests on zebrafish were performed according to the OECD Guideline for Testing of Chemicals, Fish, Acute Toxicity Test OECD 203 (1992), yet the duration of the test was prolonged to 30 days. The exposure of sexually mature individuals of zebrafish (length 2.0±1.0 cm) to the solution with or without the nanoparticles tested was performed in covered 15 L glass tanks. Then, 5 concentrations of NiO NPs were selected using a spacing factor in the range of 2.0. The highest NiO NPs concentration used was 100 mg/L. At the beginning of the experiment, the water level on each fish tank was marked and evaporated water was refilled weekly with aerated distilled water of the same temperature. The concentration of NiO NPs in the tanks was stable during the test, because NiO NPs are not biodegradable and the water volume was regularly refilled.

The number of fish individuals in control and experimental groups was 20. The ratio fish/water was 1g of fish weight to 2.0–2.2 L of water. The toxicity test was static. Control and experimental groups were kept in 24 °C water, total hardness of water was dgH=13 N°, the light-dark regimen was 12 h light/12 h darkness, oxygen concentration >60%, pH=8.3–8.6.

The LC_50_ values were calculated according to the Lichtfield-Wilcoxon method (Lichtfield & Wilcoxon, 1949).

## Results

**Figure 1:**
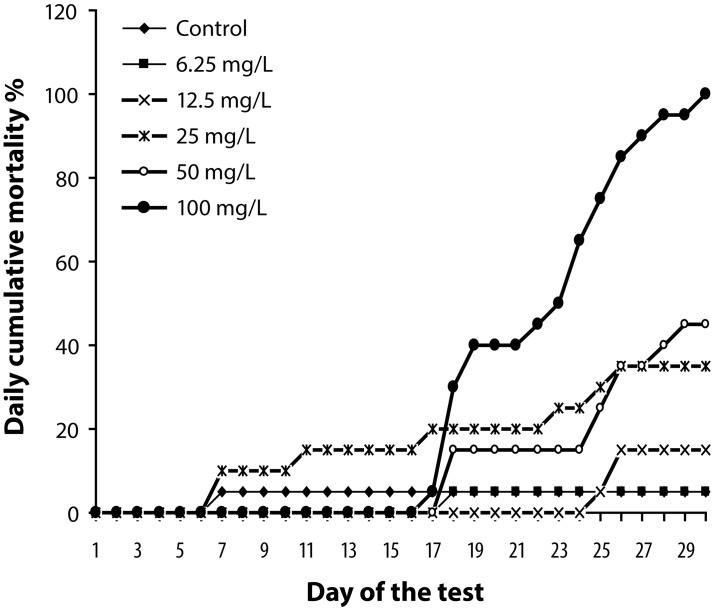
Daily cumulative mortality (in %) of different NiO NPs concentrations for sexually mature individuals of zebrafish *Danio rerio.*

The results of daily cumulative mortality of sexually mature individuals of the zebrafish *Danio rerio* exposed to different concentrations of NiO NPs are presented in Figure 1. Up to day 17 from the beginning of the exposure, the fish mortality in the highest concentration of 100 mg/L NiO NPs was the same as in controls, *i.e.* 5% (mortality in control conditions must not exceed 10% during the whole experiment). After 18 days of exposure to the concentration of 100 mg/L, the cumulative mortality started to increase rapidly until it reached values of 100% on day 30 from the beginning of exposure.

At the end of the experiment, the resulting mortality (after subtraction of 5% of the control) was: 40% (50 mg/L of NiO NPs), 30% (25 mg/L of NiO NPs), 10% (12.5 mg/L of NiO NPs) and 0% (6.25 mg/L of NiO NPs). The calculated LC_50_ value of the 30-day chronic toxicity is 45.0 mg/L (number of fish in the experiment n=120, number of fish used for calculation of LC_50_ value n=80).

The minimum dose at which a 100% mortality of experimental fish was observed during the experiment was 100 mg/L NiO NPs. The maximum dose at which no mortality was observed during 30 days was 6.25 mg/L, as the mortality did not exceed the tolerated value of 10% of fish at this dose.

In the course of the experiment, we did not observe any visible manifestanion of NiO NPs toxicity – morphological or behavioral changes in the fish.

## Discussion

On using a 96-hour acute fish toxicity test, we found in our previous experiments (Kovriznych *et al.* 2013) that according to the guideline ON 46 6807 (1988), NiO NPs belonged to the 2^nd^ class of toxicity (low toxic substances; 100–1 000 mg/L). However, by prolongation of its action on zebrafish, the toxicity increased and 30-day exposure shifted NiO NPs toxicity to the 3^rd^ class of toxicity (medium toxic substances; 10–100 mg/L) for zebrafish.

Our results are in accordance with Han *et al.*, 2012 who demonstrated in *Gracilaria lemaneiformis* that the concentration of accumulated NiO NPs increased with the extended incubation time and a considerable amount of NiO NPs remained in the bodies of *Gracilaria lemaneiformis* even after incubation. Exposure to NiO NPs led to significant reduction of cell viability, between 20–60% relative to controls. Lipid peroxidation in *Gracilaria lemaneiformis* exposed to NiO NPs was elevated compared to the control and it further increased with the incubation time. Further, at the highest concentration of NiO NPs, reactive oxygen species (ROS) levels were more than three times enhanced. Also Oberdörster *et al.* (2006) have shown that filter-feeding crustaceans (*Daphnia magna*) can accumulate nanoparticles when exposed *via* the water column.

Along with the rapid development of coastal regions, NiO NPs from welding processes becomes an important source of nano-pollution in coastal seawaters. NiO NPs may be released by direct aerial emission of particles to surface waters, leakages and spills, and indirect storm-water runoff from land (Wiesner *et al.*, 2006). Thus, pollution with NiO NPs, similarly as with other nanoparticles, poses a risk for the environment and human health as well. The toxicity of NiO NPs varies for different organisms, exhibiting the highest toxicity for algae (the 72-h EC_50_ value was 32.28 mg/L; Gong *et al.*, 2011). Microorganisms such as Escherichia coli, Bacillus subtilis, and Streptococcus aureus are less sensitive to NiO NPs (the EC_50_ value ranges from 121 to 160 mg/L; Baek and An, 2011). In comparison to mammalian animals, NiO NPs are more toxic for fish than for mammals (LD_0_=5 g/kg for rats; US Research Nanomaterials).

On balance, these findings indicate that exposure to NiO nanoparticles, especially for long periods of time, may exert a negative impact on the population of aquatic organisms and on food web dynamics in aquatic systems.

## Conclusion

Our experiments were the first to show an increase in toxicity of NiO NPs by extending the time of exposure of the adult zebrafish *Danio rerio.* These findings suggest that regardless the relatively low acute toxicity, pollution with NiO NPs, similarly as with other nanoparticles, may represent a risk for aquatic systems.
